# Long‐Term Real‐World Evaluation of Tofacitinib's Effectiveness and Safety in Japan: Insights Into Optimal Timing for Dose Reduction

**DOI:** 10.1002/jgh3.70447

**Published:** 2026-07-19

**Authors:** Yoriaki Komeda, Masashi Kono, Tomoyuki Nagai, Shunsuke Omoto, Mamoru Takenaka, Satoru Hagiwara, Naoko Tsuji, George Tribonias, Masatoshi Kudo

**Affiliations:** ^1^ Department of Gastroenterology and Hepatology, Faculty of Medicine Kindai University Osaka Japan; ^2^ Department of Gastroenterology Red Cross Hospital Athens Greece

**Keywords:** drug tapering, Janus kinase inhibitors, retrospective studies, tofacitinib, ulcerative colitis

## Abstract

**Aims:**

Tofacitinib (TOF), an oral pan‐Janus kinase (JAK) inhibitor, is a therapeutic option for moderate to severe ulcerative colitis (UC). While short‐term efficacy has been reported, its long‐term real‐world outcomes—particularly in Asian populations—and optimal dose reduction timing are incompletely understood.

**Methods and Results:**

This single‐center, retrospective study included 63 patients with UC treated with TOF between October 2018 and April 2025. Clinical outcomes—response, remission, steroid‐free remission, and adverse events—were assessed at Weeks 26, 52, and 104. The impact of dose reduction (20–10 mg/day) was analyzed, focusing on endoscopic mucosal healing (Mayo endoscopic subscore 0–1). At Week 26, clinical response and remission rates were 63% and 53%, respectively; 43% of patients achieved steroid‐free remission by Week 52, and 41% maintained it at Week 104. Early responders were associated with treatment efficacy in a lower partial Mayo score (OR = 1.724, 95% CI: 0.994–2.988, *p* = 0.052). Among 26 patients with dose reduction, relapse occurred in 5.6% with mucosal healing versus 62.5% without. Significant factors associated with relapse/exacerbation were TOF dose reduction based on clinical remission alone without endoscopic confirmation (*p* = 0.003, OR = 5.26) and disease duration (*p* = 0.019, OR = 0.86). Adverse events were infrequent and manageable, with no malignancies or deaths reported.

**Conclusion:**

TOF demonstrated sustained effectiveness and safety in UC. Mucosal healing was strongly associated with successful dose de‐escalation, representing a key treatment target. These findings support treat‐to‐target strategies incorporating endoscopic assessment to guide TOF dose reduction.

## 1 Introduction

Ulcerative colitis (UC) is a type of idiopathic inflammatory bowel disease (IBD) marked by persistent inflammation of the colonic mucosa. It is believed to stem from a complex interplay of genetic predisposition and environmental factors. Although the precise etiology remains unclear, aberrant intestinal immune responses may contribute to the onset and persistence of inflammation [1]. In recent decades, the prevalence of UC in Japan has increased markedly, nearing the levels reported in Western countries. Globally, the prevalence of UC varies widely, ranging from 2.4 to 505 cases per 100 000 individuals [1]. In Japan alone, reported prevalence rates were 18.1 per 100 000 in 1991 [2] and 172.9 per 100 000 in 2014 [3], reflecting a trend of rising incidence [1, 2, 3].

Steroids are a common treatment for UC; however, they trigger a wide range of adverse effects [1]. The ultimate goal of UC treatment is to achieve sustained steroid‐free remission and improve patients' quality of life (QOL) [4]. Current treatment strategies include 5‐aminosalicylic acid (5‐ASA) compounds, corticosteroids, immunomodulators, and advanced biologic therapies such as anti‐TNF‐α antibodies (infliximab, adalimumab, and golimumab), anti‐α4β7 integrin antibody (vedolizumab), and anti‐IL‐12/23 p40 subunit antibody (ustekinumab) [4].

Tofacitinib (TOF), an orally administered small‐molecule JAK inhibitor, was approved in Japan in 2018 for the treatment of moderate to severe UC. It offers several advantages over conventional biologic therapies, including rapid onset of action, oral administration, and the absence of immunogenicity [5]. Since its approval, the development of JAK1‐selective inhibitors such as filgotinib and upadacitinib (UPA) has further expanded the therapeutic options for UC.

Cytokines play a crucial role in maintaining intestinal homeostasis, serving as key contributors to the pathogenesis of IBD. A central pathway mediating cytokine signaling is the Janus kinase/signal transducer and activator of transcription (JAK–STAT) pathway [6]. TOF exerts its therapeutic effect by inhibiting JAKs, which, in turn, prevents the phosphorylation and nuclear translocation of STATs. Ultimately, this therapeutic effect limits the transcription and production of pro‐inflammatory cytokines.

The JAK family consists of JAK1, JAK2, JAK3, and tyrosine kinase 2 (TYK2). TOF is a nonselective JAK inhibitor showing predominant activity against JAK1 and JAK3 [7, 8]. It offers several advantages, including oral bioavailability, predictable pharmacokinetics, and lack of immunogenicity, which distinguish it from monoclonal antibody therapies. These characteristics have made TOF a promising novel therapeutic option for UC. Although several real‐world data reports have been published [9, 10, 11, 12, 13, 14], none of the studies have reported long‐term outcomes (up to 104 weeks) in Asia, with the optimal timing for TOF dose reduction remaining unclear.

In this study, we aimed to evaluate the long‐term effectiveness and safety of TOF over a 104‐week period using real‐world data from Japan. In addition, we examined the appropriate timing for dose reduction following clinical response.

## 2 Methods

This was a retrospective observational study conducted at a single tertiary center between October 2018 and April 2025. The study protocol was approved by the Kindai University Ethics Committee (Approval number: 2024‐155), and all patients were given the opportunity to opt out of participation. Consecutive patients with UC who received TOF during this period were included. The diagnosis of UC was established according to the Japanese Society of Gastroenterology guidelines, based on characteristic endoscopic and histological findings [15].

Baseline patient characteristics were collected at initiation of TOF therapy, including age, sex, disease extent (pancolitis, left‐sided colitis, or proctitis), prior use of biologics (anti‐TNFα, anti‐integrin, or anti‐IL‐12/23 antibodies), history of prednisolone exposure, and clinical parameters such as the total Mayo score, Mayo endoscopic subscore (MES), and serum C‐reactive protein (CRP) (Table 1).

**TABLE 1:** Patient characteristics at baseline.

Patient characteristics	All cases (*n* = 63)
Male sex, *n* (%)	37 (59%)
Age, years, median (IQR)	43.0 (33.0–58.0)
Disease duration, years, median (IQR)	6.0 (2.0–11.0)
Disease type	Extensive colitis: 57
	Left‐sided colitis: 6
Partial endoscopic score	
PMS, median	7.0
Mayo endoscopic subscore, *n* (%)	
MES 3	31 (49%)
MES 2	31 (49%)
MES 1	1 (2%)
Serum CRP, mg/dL, median (IQR)	0.78 (0.11–2.87)
No prior biologic treatment (naïve), *n* (%)	38 (60%)
Prior biologic treatment, *n* (%)	
1 agent	18 (29%)
2 or more agents	7 (11%)
Steroid‐refractory, *n* (%)	5 (8%)
Steroid‐dependent, *n* (%)	58 (92%)

*Note:* Baseline patient characteristics collected included age, sex, disease extent (pancolitis, left‐sided colitis, proctitis), history of biologic use (antitumor necrotic factor‐α, anti‐integrin, anti‐interleukin‐12/23 antibodies), prior use of prednisolone, and the following clinical parameters: Mayo score, endoscopic score, and C‐reactive protein.

Abbreviations: CRP, C‐reactive protein; MES, Mayo endoscopic subscore; PMS, partial Mayo score.

The primary endpoints of interest were clinical response, clinical remission, steroid‐free remission at 26, 52, and 104 weeks during the intent‐to‐treat analysis; the continuation of TOF; and the incidence of adverse events. Clinical response was defined as a reduction in the total Mayo score of at least 30% and at least three points from baseline, together with an improvement in the rectal bleeding subscore by at least one point or to a score of 0–1. Clinical remission was defined as a total Mayo score of 2 or lower with no individual subscore greater than 1. Steroid‐free remission was defined as clinical remission without concomitant corticosteroid use. TOF continuation was assessed using Kaplan–Meier analysis of drug survival. Adverse events were retrospectively extracted from medical records and included gastrointestinal symptoms, dyslipidemia, thrombosis, cytopenia, renal dysfunction, infections (herpes zoster, coronavirus disease [COVID‐19]), and malignancies.

### 2.1 TOF Treatment Protocol and Criteria for Dose Reduction

TOF was generally initiated at a dose of 20 mg/day as induction therapy in accordance with routine clinical practice at our institution. Following induction, patients who achieved clinical response or remission were put on maintenance therapy with 10 mg/day. Dose reduction from 20 to 10 mg/day was considered at the discretion of the treating physician, based on the overall clinical course, including improvement in symptoms, partial Mayo score (PMS), inflammatory markers, and, when available, endoscopic findings. In principle, dose reduction was preferentially considered in patients who had achieved stable clinical remission and/or mucosal healing. Endoscopic findings obtained within 3 months before dose reduction were reviewed, and mucosal healing was defined as an MES of 0 or 1.

In some cases, dose reduction was performed based on clinical remission alone when endoscopic reassessment was not available or when safety concerns, including infection risk during the COVID‐19 pandemic, were considered. For patients who underwent a dose reduction from 20 to 10 mg/day, endoscopic findings within 3 months of the dose change were reviewed. Mucosal healing was defined as an MES of 0 or 1.

Relapse was defined as worsening of clinical symptoms requiring treatment escalation, including an increase in the PMS, initiation or dose escalation of corticosteroids, or a change in therapeutic agents. An analysis was conducted to identify factors associated with successful dose reduction of TOF.

Univariate and multivariate analyses were conducted to identify baseline factors associated with clinical response at Week 26. Statistical analyses were performed using Python (version 3.x), with the use of the scikit‐learn and statsmodels libraries. A two‐sided significance level of 5% was applied. The primary outcome was treatment efficacy, with “response” defined as the occurrence of the event. Continuous variables were analyzed as continuous variables without categorization.

First, univariate logistic regression analyses were conducted to evaluate the association between each variable and treatment efficacy. Subsequently, multivariate logistic regression analysis (forced‐entry method) was performed, in which prior biologic exposure, CRP, MES, and PMS were simultaneously entered as explanatory variables. Odds ratios (ORs) and 95% confidence intervals (CIs) were calculated for each variable.

Drug continuation was analyzed using the Kaplan–Meier method. The time to treatment discontinuation was defined as the interval from initiation of TOF to treatment discontinuation for any reason, including loss of response, adverse events, or other clinical decisions. Patients who continued treatment at the last follow‐up were censored at that time point.

## 3 Results

### 3.1 Clinical Effectiveness

**FIGURE 1:**
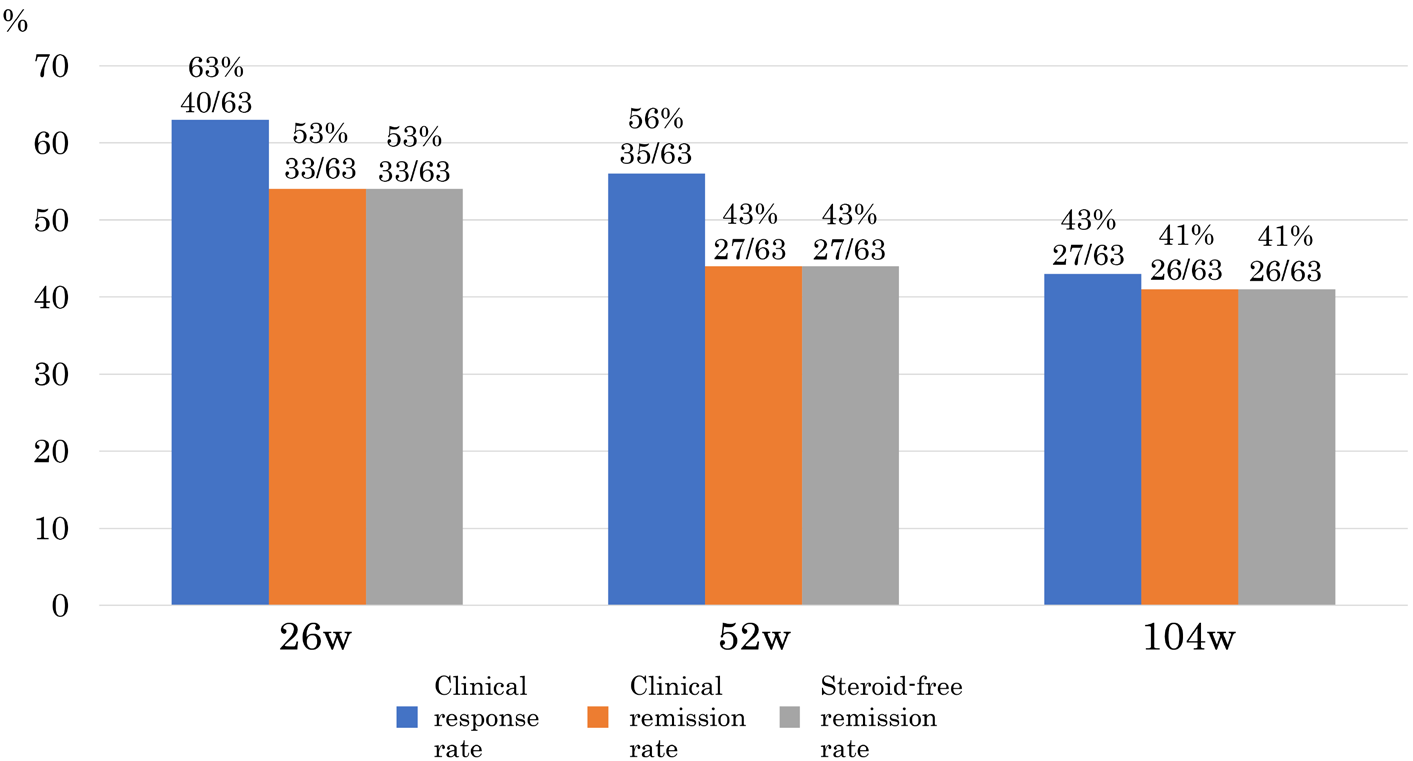
Clinical response rate, clinical remission rate, and steroid‐free remission rate with TOF. Bar graphs showing clinical response rate, clinical remission rate, and steroid‐free remission rate at 26 weeks, 52 weeks, and 104 weeks. TOF, Tofacitinib.

**FIGURE 2:**
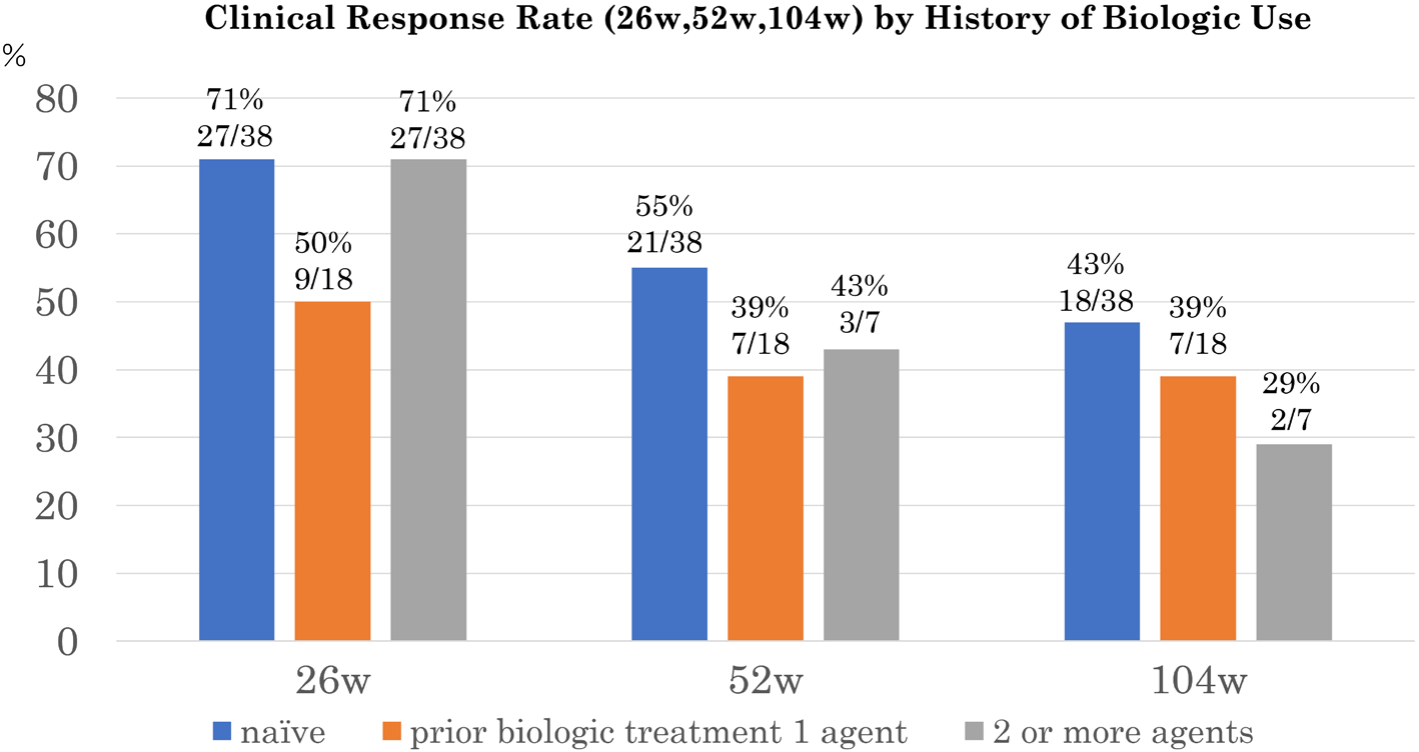
Clinical response rate (26 weeks) by history of biologic use. Bar graph showing groups stratified by treatment history: Biologic‐naïve, single biologic, and two or more biologics. TOF demonstrated favorable efficacy even in patients with prior biologic exposure. TOF, tofacitinib.

The clinical response and remission rates at Weeks 26, 52, and 104 after initiating TOF are shown in Figure 1. A CONSORT‐style patient flowchart summarizing patient inclusion, clinical outcomes, dose reduction, and relapse after dose reduction is shown in Figure S1. At Week 26, the clinical response rate was 71% in biologic‐naïve patients and 56% in those with prior biologic use, as shown in Figure 2.

**FIGURE 3:**
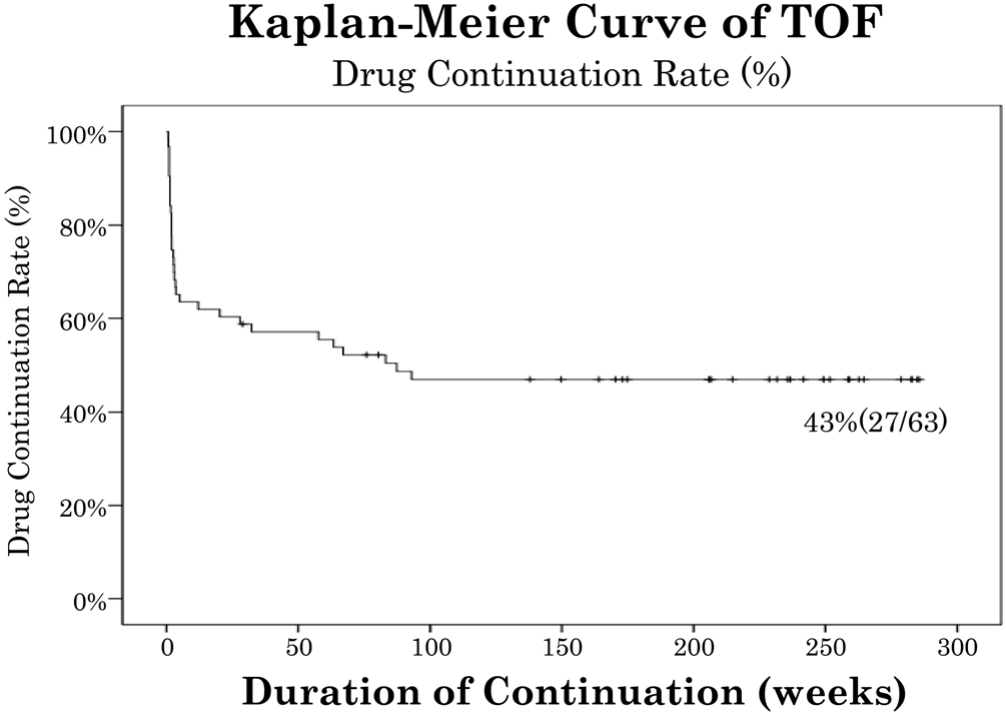
Kaplan–Meier curve. Patients who exhibited an early clinical response to TOF were more likely to maintain long‐term remission. The continuation rate of TOF at Week 104 was 41%. TOF, tofacitinib.

According to Fisher's exact test, the *p* = 0.283, indicating no statistically significant difference between biologic‐naïve patients and those with prior biologic use. The steroid‐free remission rate at Week 52 was 43%. At Week 104, the cumulative remission rate among patients who had achieved remission was 43% (27/63), as shown in Figure 3.

Univariate and multivariate analyses were conducted to identify factors associated with clinical response at Week 26 (Table 2). In this study, a total of 63 patients were included in the analysis. Univariate logistic regression analysis showed that none of the variables were significantly associated with treatment efficacy. The results were as follows: prior biologic exposure (OR: 0.879, 95% CI: 0.454–1.699, *p* = 0.701), CRP (OR: 0.943, 95% CI: 0.816–1.088, *p* = 0.421), MES (OR: 0.878, 95% CI: 0.329–2.339, *p* = 0.794), and PMS (OR: 1.131, 95% CI: 0.854–1.498, *p* = 0.392).

**TABLE 2:** Baseline characteristics associated with clinical response of tofacitinib at Week 26: Univariate and multivariate analyses.

Analysis of factors associated with treatment efficacy (*n* = 63)						
Variables	Univariate OR	95% CI	*p*	Multivariate OR	95% CI	*p*
Number of prior biologics	0.879	0.454–1.699	0.701	0.796	0.386–1.638	0.535
CRP	0.943	0.816–1.088	0.421	0.915	0.780–1.074	0.277
MES	0.878	0.329–2.339	0.794	0.201	0.029–1.393	0.104
PMS	1.131	0.854–1.498	0.392	1.724	0.994–2.988	0.052

Abbreviations: CI, confidence interval; CRP, C‐reactive protein; MES, Mayo endoscopic score; OR, odds ratio; PMS, partial Mayo score.

In the multivariate logistic regression analysis (forced‐entry method), none of the variables achieved statistical significance. PMS showed a trend toward an association with treatment efficacy (OR: 1.724, 95% CI: 0.994–2.988, *p* = 0.052).

### 3.2 Adverse Events Associated With TOF

Adverse events observed during TOF treatment are summarized in Table 3. Overall, the incidence of adverse events was low, and no cases of malignancy or death were reported. Known safety concerns, including herpes zoster and thrombosis, were observed but remained manageable through appropriate monitoring and timely intervention. These findings suggest that TOF maintains a generally favorable safety profile in this patient population (Table 3).

**TABLE 3:** Incidence rate of adverse events, *n* (%).

Safety profile	Incidence number (rate)
Gastrointestinal symptoms	4 cases (6.3%)
Dyslipidemia	4 cases (6.3%)
Thrombosis	2 cases (3.2%)
Cytopenia	1 case (1.6%)
Renal dysfunction	1 case (1.6%)
Herpes zoster	3 cases (4.8%)
Coronavirus disease (COVID‐19)	1 case (1.6%)
Malignancy	None
Death	None

*Note:* In terms of safety, adverse events were generally manageable, and serious complications were avoided through appropriate monitoring and preventive interventions, particularly for herpes zoster and thrombosis.

### 3.3 Impact of Dose Reduction and Relationship With Mucosal Healing (MES 0–1) in Table 4

**TABLE 4:** Factors associated with relapse after tofacitinib dose reduction in patients with ulcerative colitis.

Mucosal healing	Number of no relapse	Number of relapse
MES0	12	0
MES1	5	1

*Note:* Univariate and multivariate logistic regression analyses evaluating predictors of relapse following dose reduction of tofacitinib (TOF) from 20 to 10 mg/day in patients who achieved clinical remission. Variables assessed included dose reduction strategy, disease activity indices, prior biologic exposure, and disease duration.

Of the 26 patients who achieved clinical remission, the TOF dose was reduced from 20 to 10 mg/day. Following dose reduction, six patients (6/26, 23%) experienced a clinical relapse. Among these, five (5/6, 83%) responded again to re‐escalation of TOF, while one of those achieved remission after switching to UPA.

Endoscopic mucosal healing (defined as MES 0–1) was achieved in 18 patients, of whom only 1 (1/18, 5.6%) experienced relapse. In contrast, among the eight patients without mucosal healing, five (5/8, 62.5%) relapsed (Table 4). Fisher's exact test yielded a *p* = 0.0045, indicating a statistically significant difference in relapse rates based on mucosal healing status. These findings suggest that the risk of relapse is significantly lower in patients who achieve mucosal healing. Among the six cases of disease exacerbation, five (83%) regained clinical response after TOF re‐escalation. The remaining patient, who did not respond to re‐escalation, achieved remission after switching to UPA.

Although our sufficiently powered multivariate analysis was limited due to the small sample size (*n* = 26), we conducted additional analyses considering key baseline factors (dose reduction based on clinical remission alone without endoscopic confirmation, prior biologic exposure, disease activity, and disease duration) to evaluate the potential influence of residual confounding.

In the univariate analysis, dose reduction based on clinical remission alone, without endoscopic confirmation, was significantly associated with relapse (*p* = 0.002, OR = 5.88); similarly, disease duration was also associated with relapse (*p* = 0.041, OR = 0.89). In contrast, PMS and the number of prior biologics were not significantly associated with relapse in Table 4.

In the multivariate analysis, dose reduction based on clinical remission alone (without endoscopic confirmation) remained an independent risk factor associated with relapse (*p* = 0.003, OR = 5.26). Disease duration was also independently associated with relapse (*p* = 0.019, OR = 0.86) in Table S1.

Significant factors associated with relapse or exacerbation after TOF dose reduction were identified as dose reduction based on clinical remission alone without endoscopic confirmation (*p* = 0.003, OR = 5.26) and disease duration (*p* = 0.019, OR = 0.86).

Notably, although no statistically significant difference in relapse rates was observed between MES 0 and MES 1 (*p* = 0.333, Fisher's exact test), no relapse occurred within the MES 0 group. Consequently, the small sample size may have resulted in insufficient statistical power to detect a difference.

The Kaplan–Meier analysis demonstrates the long‐term drug persistence of TOF over time. The overall continuation rate was 43% (27/63), indicating that less than half of the patients were able to maintain long‐term treatment (Figure 3).

Patients who achieved an early clinical response tended to maintain TOF treatment for a longer duration, suggesting that initial therapeutic response may be predictive of sustained treatment continuation.

## 4 Discussion

In this study, we evaluated the long‐term effectiveness, safety, and feasibility of a dose reduction strategy for TOF in patients with UC, based on the 104‐week real‐world data in Japan.

### 4.1 Long‐Term Effectiveness and Predictive Factors

At Weeks 26, 52, and 104, the clinical response, remission, and steroid‐free remission rates were 63% 40/63, 53% 33/63, 53% 33/63; 56% 35/63, 43% 27/63, 43% 27/63; and 43% 27/63, 41% 26/63, 41% 26/63, respectively. At each time point, remission was maintained in over 40% of patients. These outcomes are consistent with results from Phase III clinical trials, including the OCTAVE trial [5] and the RIVETING study [16], supporting the utility of TOF as a long‐term maintenance therapy. When stratified by prior biologic exposure, the highest clinical response rate was observed in biologic‐naïve patients (71%), suggesting that TOF may be an effective first‐line option in patients without prior exposure to biologics. Moreover, patients who achieved a clinical response by Week 26 demonstrated higher drug continuation rates, indicating that early therapeutic success may predict long‐term maintenance.

Furthermore, patients who achieved a clinical response by Week 26 demonstrated significantly higher drug continuation rates, suggesting that early treatment response may be associated with favorable long‐term outcomes. This observation is consistent with previous clinical trials and real‐world studies of TOF, in which early responders were more likely to achieve sustained remission and continued treatment success [5, 11].

Although no statistically significant difference in clinical response at Week 26 was observed between biologic‐naïve patients and those with prior biologic exposure (*p* = 0.283), this result should be interpreted with caution. The absence of statistical significance may be attributable to the limited sample size and consequent insufficient statistical power, particularly within the subgroup of biologic‐experienced patients.

Notably, the observed response rate was numerically higher in biologic‐naïve patients (71%) than in those with prior biologic exposure (56%), suggesting a potential trend that did not reach statistical significance. This tendency is consistent with previous studies reporting reduced treatment efficacy in biologic‐experienced populations [12, 13].

Therefore, larger studies are warranted to clarify whether prior biologic exposure significantly impacts the effectiveness of TOF.

Notably, a relatively high response rate (5/7) was observed even in patients who had failed two or more prior biologic therapies. Similar findings have been reported in biologic‐experienced and hospitalized patients with severe UC treated with TOF, suggesting its potential effectiveness even in difficult‐to‐treat populations [17]. This finding may be explained by the distinct mechanism of action of TOF compared with conventional biologic agents. While biologics typically target single cytokines or adhesion molecules, TOF broadly inhibits multiple cytokine signaling pathways through JAK–STAT inhibition, potentially overcoming resistance to prior biologics.

In addition, the rapid onset of action and lack of immunogenicity may contribute to its effectiveness in treatment‐refractory patients. These findings suggest that TOF may represent a valuable therapeutic option even in patients with limited treatment alternatives. However, due to the small sample size, this observation should be interpreted with caution and warrants further validation in larger cohorts.

In this study, logistic regression analysis was performed to investigate factors associated with treatment efficacy; however, no statistically significant independent factors were identified in the multivariate analysis. Meanwhile, PMS showed a trend toward an association with treatment efficacy (*p* = 0.052). Since no significant association was observed in the univariate analysis, this divergence suggests that adjusting for other variables may have revealed a potential independent association for PMS.

When compared with previous real‐world data from Asia, our results appear broadly consistent but provide several novel insights. A recent multicenter study from Japan reported clinical remission rates of approximately 40%–50% at 52 weeks [13], which is congruent with our 43% remission rate at the same time point. Other Japanese real‐world studies evaluating advanced therapies in hospitalized UC patients have also demonstrated favorable effectiveness and safety profiles across multiple treatment modalities [18]. Similarly, other Asian real‐world cohort studies demonstrated sustained effectiveness of TOF, particularly in biologic‐experienced populations [12].

Notably, our study extends these findings by providing outcomes at 104 weeks, which remain scarce regarding Asian populations. Significantly, remission was maintained in over 40% of patients even at the 104‐week mark, suggesting durable long‐term effectiveness in a real‐world setting.

Furthermore, while previous Asian studies have primarily focused on short‐ to mid‐term outcomes, our analysis highlights the clinical relevance of endoscopic mucosal healing as a determinant of successful dose reduction. This aspect has been insufficiently addressed in prior Asian cohorts, where treatment optimization strategies such as dose de‐escalation have not been systematically evaluated.

### 4.2 Safety and Adverse Event Management

Adverse events observed during TOF treatment included gastrointestinal symptoms (6.3%), dyslipidemia (6.3%), thrombosis (3.2%), cytopenia (1.6%), renal dysfunction (1.6%), herpes zoster (4.8%), and COVID‐19 infection (1.6%). No malignancies or deaths occurred. These rates align with prior findings [10], and TOF demonstrated a generally favorable safety profile under appropriate monitoring. Notably, the risk of herpes zoster was mitigated through early dermatological referral and patient education. Furthermore, thrombotic events were detected early through D‐dimer elevation and managed promptly, suggesting that risks commonly associated with JAK inhibitors can be effectively controlled via proactive clinical monitoring.

The cumulative incidence of adverse events was comparable to previous reports, and treatment discontinuation occurred only in cases of thrombosis, cytopenia, or renal dysfunction. Herpes zoster was manageable through dermatological consultation and antiviral therapy. Thrombosis was detected early by regular monitoring, including D‐dimer testing, and successfully managed with anticoagulation, preventing progression to severe events. These findings suggest that, in real‐world practice, long‐term TOF therapy can be conducted safely with appropriate monitoring and preventive strategies.

### 4.3 Dose Reduction Strategy and the Significance of Mucosal Healing

Among 26 patients who underwent dose reduction from 20 to 10 mg/day, those who achieved mucosal healing (MES 0–1; *n* = 18) had a relapse rate of only 5.6% (1/18), compared to 62.5% (5/8) in those without mucosal healing (*n* = 8). This difference was statistically significant (*p* = 0.0045, Fisher's exact test), indicating that achieving mucosal healing is an important predictor of successful dose reduction. These findings are consistent with the RIVETING trial [16], which suggests that patients in deep endoscopic remission are more likely to maintain remission after dose reduction. Furthermore, 83% (5/6) of relapsed patients responded to TOF re‐escalation, indicating the feasibility of re‐induction as a practical postreduction strategy. The results highlight the importance of mucosal healing in a treat‐to‐target strategy [19]. We identified dose reduction based on clinical remission alone (without endoscopic confirmation) and disease duration as significant factors associated with relapse after TOF dose reduction. One patient who did not show a clinical response after TOF re‐escalation achieved remission following a switch to UPA. Switching from TOF to UPA after loss of TOF efficacy has been reported to be effective [20]. Overall, in cases of relapse following TOF dose reduction, TOF re‐escalation demonstrated high therapeutic efficacy.

In this study, we defined mucosal healing as an MES of 0–1, which is consistent with the definition used in many previous clinical trials and real‐world studies of TOF. However, we acknowledge that recent literature has distinguished MES 0 (complete mucosal healing) from MES 1 (mild residual erythema), and some reports suggest that outcomes may differ between these two groups. Due to the limited sample size in our cohort, MES 0 and 1 were combined to maintain statistical feasibility; however, we agree that future studies should separately evaluate these categories to clarify whether MES 0 provides additional prognostic value over MES 1 in predicting long‐term remission and successful dose reduction.

### 4.4 Limitations and Future Directions

This study was a single‐center retrospective analysis, with inherent limitations including a small sample size and variability in observation periods. Notably, defining the timing of endoscopic assessment before dose reduction as “within 3 months” introduced some inconsistency. Considering the rapid onset of action of TOF, this assessment window may be too broad. Furthermore, the study period coincided with the COVID‐19 pandemic, which likely introduced selection bias regarding dose reduction strategies. Consequently, in many cases where clinical remission was achieved without mucosal healing, early dose reduction was implemented to mitigate the risk of infection.

Furthermore, regarding the combined analysis of MES 0 and MES 1, no statistically significant difference in relapse rates was observed between the two groups (*p* = 0.333, Fisher's exact test). However, it is noteworthy that no relapse events were observed in the MES 0 group, and the small sample size may have resulted in inadequate statistical power to detect a clinical difference.

More stringent protocol definitions are needed in future studies. From a preventive perspective, vaccination against herpes zoster may be considered as part of general risk management strategies in patients receiving JAK inhibitor therapy, based on existing clinical recommendations.

## 5 Conclusions

The findings of this study demonstrate that TOF is an effective and safe therapeutic option for patients with refractory UC. In particular, patients who exhibited an early clinical response to TOF were more likely to maintain long‐term remission. Regarding dose reduction, the relapse rate was significantly lower in patients who had achieved endoscopic mucosal healing (MES 0–1). These results indicate that mucosal healing may serve as a critical indicator for determining the timing of dose de‐escalation. Moreover, re‐escalation of TOF upon relapse resulted in a high re‐induction rate, demonstrating its utility as a reliable rescue strategy. In terms of safety, adverse events were generally manageable, and serious complications were avoided through vigilant monitoring and preventive interventions, particularly for herpes zoster and thromboembolism. Taken together, TOF represents a promising treatment strategy for UC, especially when patient selection and management are meticulously tailored.

## Funding

The authors have nothing to report.

## Ethics Statement

This was a retrospective observational study conducted at a single tertiary center between October 2018 and April 2025. The study protocol was approved by the Kindai University Ethics Committee (Approval number: 2024‐155).

## Consent

All patients were given the opportunity to opt out of participation.

## Conflicts of Interest

Yoriaki Komeda has received lecture honoraria from pharmaceutical companies related to tofacitinib (TOF), which constitutes a potential conflicts of interest. The other authors declare no conflicts of interest.

## Supporting information


**Figure S1:** Patient flowchart (CONSORT format).
**Table S1:** Analysis of factors associated with relapse (*n* = 26).

## Data Availability

The data that support the findings of this study are available from the corresponding author upon reasonable request.
