# Supplementary material for: Long‐Term Real‐World Evaluation of Tofacitinib's Effectiveness and Safety in Japan: Insights Into Optimal Timing for Dose Reduction
Source: JGH Open. 2026 Jul 19;10(7):e70447. doi: 10.1002/jgh3.70447 (PMC13381722; doi:10.1002/jgh3.70447)
Supplement: Supplementary file 1 — Figure S1: Patient flowchart (CONSORT format). Table S1: Analysis of factors associated with relapse (n = 26). [file JGH3-10-e70447-s001.docx]

**Supplementary Material**

Assessed for eligibility (n = 63)

↓

Included in analysis (n = 63)

↓

Week 26: Clinical response, 40 (63%); Clinical remission, 33 (53%); Steroid-free remission, 33 (53%)

↓

Week 52: Clinical response, 35 (56%); Clinical remission, 27 (43%); Steroid-free remission, 27 (43%)

↓

Week 104: Clinical response, 26 (43%); Clinical remission, 26 (41%); Steroid-free remission, 26 (41%)

↓

Dose reduction (n = 26): Relapse (n = 6)

↓

Mucosal healing: 1/18 (5.6%) vs 5/8 (62.5%)

**Supplementary Figure S1**. Patient flowchart (CONSORT format)

**Supplementary Table S1**. Analysis of factors associated with relapse (n = 26)

| **Variables** | **Univariate analysis** | | | | **Multivariate analysis** | | | |
| --- | --- | --- | --- | --- | --- | --- | --- | --- |
|  | P | OR | 95% CI | | P | OR | 95%CI | |
| Dose reduction based on clinical remission alone (without endoscopic confirmation) | 0.002 | 5.88 | 2.85 | 11.20 | 0.003 | 5.26 | 2.64 | 10.36 |
| Partial Mayo score (PMS) | 0.610 | 1.05 | 0.91 | 1.23 | 0.928 | 1.01 | 0.85 | 1.28 |
| Number of prior biologics | 0.490 | 1.48 | 0.52 | 3.98 | 0.723 | 1.76 | 0.51 | 0.72 |
| Disease duration | 0.041 | 0.89 | 0.71 | 0.98 | 0.019 | 0.86 | 0.65 | 0.09 |
